# Prevalence and severity of premenstrual tension syndrome and its impact on academic performance among female university students in Bangladesh: a cross-sectional study

**DOI:** 10.1186/s12889-026-28124-3

**Published:** 2026-07-02

**Authors:** Md. Mahedi Hassan Lemon, Sabbir Ahmed, Mst. Khadija Khatun, Md. Shohel Rana

**Affiliations:** Department of Statistics and Data Science, Islamic University, Kushtia, 7003 Bangladesh

**Keywords:** Premenstrual tension syndrome, Premenstrual dysphoric disorder, Academic performance, Female students, Bangladesh

## Abstract

**Background:**

Premenstrual tension (PMT) syndrome is an important condition that affects women’s psychological, physical, and behavioral well-being, as well as their academic performance. This study investigates the association between premenstrual tension (PMT) symptom severity and academic performance among female university students.

**Methods:**

A cross-sectional study was conducted over an 8-month from January to August 2025 among 273 female students of Islamic University, Bangladesh. A reliable, self-administered, bilingual questionnaire was developed based on the Diagnostic and Statistical Manual of Mental Disorders, Fifth edition (DSM-5) criteria to collect the primary data. It was used to assess the prevalence and severity of psychological, behavioral, and physical symptoms as well as their impact on academic performance. Data were analyzed using SPSS (version 25.0) and Python (Pandas, NumPy, SciPy, and Matplotlib). The Chi-square test was used to examine the association between PMT and academic outcomes.

**Results:**

A total of 273 participants were selected from 976 eligible female students. The age of the participants ranged from 17 to ≥ 25 years, with a median age category of 23–24 years. The prevalence of premenstrual tension (PMT) symptoms was 65.2%, while 8.1% of participants met the criteria for premenstrual dysphoric disorder (PMDD). Severe mood swings (39.6%) and irritability (37.0%) were identified as the most frequently reported symptoms. A statistically significant association was observed between PMT and academic performance indicators, including difficulty concentrating in class (69.6%, *p* = 0.013), negative impact on examination performance (64.4%, *p* = 0.012), and difficulty participating in extracurricular activities (60.0%, *p* = 0.009). Overall, 54.9% of the female university students reported that their academic performance was negatively affected by premenstrual symptoms.

**Conclusion:**

Our study revealed a high prevalence of premenstrual tension (PMT) and premenstrual dysphoric disorder (PMDD) among female university students, with severe psychological symptoms such as mood swings and irritability negatively affecting concentration, examination performance, and participation in extracurricular activities. The findings underscore the importance of raising awareness, implementing targeted mental health support, and providing appropriate interventions to improve academic performance and overall well-being.

**Supplementary Information:**

The online version contains supplementary material available at https://doi.org/10.1186/s12889-026-28124-3.

## Background

 Premenstrual tension (PMT) syndrome is a common condition affecting women during the menstrual cycle [1]. Most women of reproductive age experience one or more emotional or physical symptoms in the premenstrual phase, which may range from mild changes to the broader spectrum of premenstrual syndrome (PMS) or the more severe form known as premenstrual dysphoric disorder (PMDD) [2]. It is estimated that nearly 75% of premenopausal women report experiencing at least one emotional or physical symptom during the two weeks before their period [3]. Premenstrual syndrome (PMS) is characterized by recurring physical, emotional, and behavioral symptoms that consistently arise during the luteal phase of the menstrual cycle and generally subside soon after menstruation begins, disappearing entirely in the follicular phase. Common manifestations include mood swings, irritability, fatigue, bloating, headaches [4]. In contrast, between 3% and 8% of reproductive-aged women experience PMDD, the most severe form of PMS. It is largely characterized by psychological difficulties that interfere with social relationships, routine functioning, and academic or occupational performance [5, 6]. While existing literature has reported associations between premenstrual symptoms and functional outcomes, there is still limited context-specific evidence on how these symptoms relate to measurable academic performance across different institutional and regional settings. This highlights the importance of further empirical studies in diverse populations.

PMT including PMS and PMDD, arises from a complex, multifactorial interplay of biological, psychological, and social factors. Consequently, identifying the exact causes of PMT remains highly challenging. Biologically, women with PMDD show heightened sensitivity to normal cyclical fluctuations of estrogen and progesterone, which influences neurotransmitter systems such as serotonin, leading to emotional and physical symptoms. Moreover, genetic differences may increase an individual’s likelihood of experiencing PMT symptoms. Psychologically, factors such as emotional distress, pre-existing mental health conditions, and reduced coping capacity can exacerbate symptoms including anxiety, depressed mood, irritability, and difficulties in concentration. Socially, cultural expectations, stigma, and lack of social support may further affect the experience of PMS/PMDD. Therefore, PMT is not a simple hormonal disorder but a multidimensional condition, where the interplay of these factors determines symptom severity and functional impairment [7–9].

The Diagnostic and Statistical Manual of Mental Disorders, Fifth Edition (DSM-5), published by the American Psychiatric Association, classifies PMDD as a distinct depressive disorder and provides the specific criteria for its diagnosis. Based on the DSM-5 criteria, in most menstrual cycles PMDD is diagnosed when an individual experiences at least five of the eleven specified symptoms that generally occur during the late luteal phase, become noticeable in the week before menstruation, start to improve within a few days after the period begins, and remain minimal or absent in the post-menstrual phase. These symptoms must involve at least one emotional or mood-related sign, such as irritability, mood swings, depression, or anxiety. They are often accompanied by other physical or behavioral indicators, including reduced interest in daily activities, difficulty focusing, fatigue, changes in appetite or sleep patterns, feelings of being overwhelmed, and physical complaints such as breast tenderness or bloating [10–12]. The symptoms should lead to considerable discomfort or disruption in everyday life and cannot be simply an exacerbation of another existing disorder [13].

Premenstrual Tension (PMT) is considered an important key factor that increasingly influences the academic life of female students. While a considerable proportion of previous research has focused on medical or health-related students, comparatively limited evidence is available regarding female students from general university settings. Academic environments in general higher education institutions may differ in terms of curriculum structure, workload, and institutional support systems, which could influence how premenstrual symptoms are experienced and managed. Evidence suggests that PMS symptoms, including emotional instability, anxiety, concentration difficulties, low mood, and fatigue during the premenstrual phase, can reduce students’ motivation and participation in academic and cognitive activities. Moreover, cognitive difficulties, including poor concentration, forgetfulness, and mental fatigue, can interfere with class attendance, attention in lectures, study performance, and the timely completion of academic assignments. Behavioral changes, such as sleep disturbances and decreased energy, along with physical symptoms including headaches, muscle pain, and general discomfort, can lead to absenteeism and limit students’ ability to complete tasks, thereby reducing overall learning time. Furthermore, the extra burden of PMS intensifies stress and anxiety, and accompanying psychological symptoms, such as mood changes and depressive feelings, may lower motivation and self-confidence, making it more difficult for students to stay engaged with their studies and maintain strong academic performance. Students with severe PMS frequently report diminished interest in studies and reduced participation in academic activities, including attendance, practical work, and group assignments. Understanding this link is essential for developing supportive interventions within educational institutions [14–18].

Despite extensive global research on premenstrual tension (PMT) and its associated symptoms, evidence on how PMT affects the academic performance of female university students remains geographically and contextually limited. While international studies suggest that premenstrual symptoms can negatively affect emotional well-being, cognitive functioning, and daily academic activities, these findings cannot be directly generalized to the sociocultural and educational context of Bangladesh.

For instance, studies from settings such as the United Arab Emirates have reported significant negative associations between PMT symptoms and academic performance [1]. However, differences in sociocultural environment, menstrual health awareness, academic systems, and access to healthcare services limit their direct applicability to Bangladeshi university students. Moreover, most existing studies have focused on medical or health-related students, leaving female students in general university disciplines comparatively underrepresented.

To the best of our knowledge, no study in Bangladesh has simultaneously examined both the prevalence and severity of PMT, along with its association with academic performance, among non-medical female university students.

Female students in Bangladesh may experience distinct social and institutional challenges that shape both the severity of premenstrual symptoms and their academic consequences. However, empirical evidence remains limited on how PMT-related emotional, cognitive, and physical symptoms influence academic outcomes in this population.

In particular, there is a lack of research exploring how PMT affects key academic indicators such as class attendance, concentration, lecture engagement, examination performance, and overall learning ability among Bangladeshi university students. Therefore, context-specific empirical evidence is needed.

This study addresses this gap by investigating the prevalence and severity of PMT and its association with academic performance among female university students at Islamic University, Kushtia, Bangladesh. The findings are expected to inform culturally appropriate interventions, student support strategies, and institutional policies to improve academic well-being.

## Methods

### Study design and participants

This descriptive cross-sectional study was conducted among female students at Islamic University, Kushtia-7003, Bangladesh, over an 8-month period from January to August 2025. The study included female students from all faculties residing in the three on-campus female dormitories: Begum Khaleda Zia Hall, Ummul Mu’minneen Aisha Siddiqa Hall, and July-36 Hall. Data were collected across different age groups, academic years, faculties, class attendance rates, and academic stress levels to ensure sample diversity and strengthen representativeness.

The study was implemented in several phases to ensure methodological rigor. The initial phase (January to February 2025) involved questionnaire development, protocol finalization, and institutional ethical approval. The active data collection phase was conducted over a defined three-month period from March to May 2025, aligned with the university academic calendar to facilitate participant accessibility. The remaining phase (June to August 2025) was dedicated to data cleaning, statistical analysis, and manuscript preparation.

Inclusion criteria were: age 17 years or older, regular menstrual cycle during the previous six months, willingness to participate, completion of at least one academic semester, and no history of death among first-degree relatives within the past six months. Exclusion criteria included diagnosed psychiatric disorders, polycystic ovarian disease, diabetes, hypertension, heart disease, other chronic medical illnesses, current pregnancy or breastfeeding, and use of hormonal contraceptives, antidepressants, or other psychotropic medications.

The sample size was determined using Cochran’s formula for finite populations [19]. The initial sample size for an infinite population was computed as:

$$\begin{aligned}\:{n}_{0}=\frac{{Z}^{2}\hspace{0.17em}p\left(1-p\right)}{{e}^{2}}\end{aligned}$$

where $\:\mathrm{Z}=1.96\:$(95% confidence level), * p=0.50* (50% response distribution), *e* = 0.05 (5% margin of error). This yielded an initial sample size of $\:{n}_{0}=384$. Since the target population was finite (* N=976*), the finite population correction was applied:

$$\begin{aligned}\:n=\frac{{n}_{0}}{1+\frac{{n}_{0}-1}{N}}\end{aligned}$$

Accordingly, the minimum required sample size was 276 participants.

A consecutive sampling technique [19] was employed instead of simple random sampling due to practical feasibility and the sensitive nature of premenstrual health-related data collection. Given the personal context of menstrual health reporting, where random selection could increase the likelihood of non-response or social desirability bias, this approach facilitated voluntary participation and maintained confidentiality during data collection within the university setting. Eligible female students residing in the selected dormitories were approached sequentially, and those who met the inclusion criteria and provided informed consent were enrolled in the study. To enhance the diversity of the recruited sample, participants were recruited across all faculties and multiple academic years within the three residential halls, thereby improving coverage across different academic subgroups and reducing the likelihood of over-representation from any single group. However, as a non-probability sampling approach was used, the possibility of selection bias cannot be entirely excluded.

A total of 281 participants were initially recruited. Among them, eight participants did not complete the questionnaire, resulting in a final analytical sample of 273 participants with complete responses.

### Data collection

An anonymous, self-administered bilingual (Bangla and English) questionnaire was employed to assess participants’ socio-demographic characteristics and the patterns and severity of premenstrual tension (PMT) symptoms. The questionnaire was developed by the authors to evaluate the prevalence, severity, and impact of PMT symptoms on academic performance among female university students, and a complete English version of the questionnaire is provided as Supplementary File 1.

The instrument was initially developed in English and subsequently translated into Bangla by the research team to ensure conceptual and linguistic equivalence. Content validity was established through expert review by researchers in public health and epidemiology, who evaluated item clarity, relevance, and cultural appropriateness, leading to minor refinements prior to data collection. Construct validity was supported through alignment of the symptom measurement scale with DSM-5 criteria for Premenstrual Dysphoric Disorder (PMDD).

Participants were instructed to report symptoms specifically from their most recent menstrual cycle, focusing on the premenstrual phase (typically 7–14 days prior to the onset of menstruation), when PMT/PMS symptoms are most prominent. To minimize recall bias and improve temporal accuracy, data collection was synchronized with participants’ ongoing menstrual cycle. Respondents completed the questionnaire either during the late luteal phase or within the first three days of menstruation while reflecting on the immediately preceding premenstrual period. This near-real-time cycle-specific reporting approach was adopted to enhance the validity and precision of symptom assessment and reduce potential misclassification associated with retrospective reporting.

The questionnaire comprised four sections. The first section gathered demographic data, including age, marital status, nationality, and year of study. The second section focused on menstrual history, recording the age at menarche (categorized as < 12 years or ≥ 12 years), the presence of PMT, and self-reported symptom severity, ranging from mild to severe.

The third section utilized a symptom rating scale aligned with the DSM-5 criteria for PMDD. This scale assessed 11 primary symptom domains, including physical/behavioral domains (e.g., lack of energy, feeling overwhelmed or out of control, changes in eating and sleeping habits, somatic symptoms including breast tenderness, bloating etc.) and psychological domains (e.g., mood swings, anxiety, irritability, concentration difficulties, depressed mood and decreased interest in usual activities). Participants rated their symptoms on a four-point scale (0 = No, 1 = Mild, 2 = Moderate, and 3 = Severe), with an additional domain evaluating the social impact of these symptoms.

The fourth section assessed the academic impact of PMT using a binary format (Yes = 1 and No = 0), focusing on class attendance, assignment submission, participation in physical and extracurricular activities, exam performance, in-class concentration and overall academic performance. The total PMT score ranged from 0 to 36 [20], categorized into four levels: Normal (0–10), Mild (11–18), Moderate (19–27), and Severe (≥ 28) [13]. The questionnaire demonstrated strong validity and reliability, confirmed by Cronbach’s alpha analysis (*N* = 273), with high internal consistency (α = 0.878 for PMT symptoms and α = 0.803 for academic impact), indicating robust measurements of PMT severity.

### Statistical analysis

All collected data were first entered into IBM SPSS Statistics version 25.0 and numerically coded (e.g., Yes = 1, No = 0) for statistical analysis. Subsequently, data were cleaned in SPSS by checking variable structure, duplicates, coding errors, and missing values to ensure accuracy and consistency. The cleaned dataset was then exported to Python (v3.12) in Google Colab and analyzed using Pandas, NumPy, SciPy, and Matplotlib for processing and statistical analysis. Descriptive statistics (frequencies, percentages) summarized socio-demographics and PMT prevalence, with a mean age of 23.23 ± 1.76 years. Furthermore, stacked bar charts were used to analyze and visualize the severity distribution of premenstrual symptoms and their impact on various domains of academic performance. The Chi-square test was conducted to examine the association between PMT (Yes/No) and academic performance. The analysis considered multiple indicators, including examination results, assignment submission, class attendance, and attentiveness during class lectures, participations in extracurricular activities, and physical activities. Finally, all analyses were performed using two-tailed tests, with statistical significance defined at p-value < 0.05.

## Results

### Characteristics of the study participants

Initially, 281 female students were approached for participation. Among them, 273 were returned valid questionnaires and included in the final analysis. The median age category of the participants was 23–24 years. Participants were grouped into four age categories ranging from 17 to 20 years to ≥ 25 years. Regarding menstrual history, the majority of participants reported menarche at ≥ 12 years (87.9%), whereas 12.1% experienced menarche before the age of 12. In terms of marital status, most respondents were single (*n* = 239, 87.5%), while 34 (12.5%) were married. The study was overwhelmingly Bangladeshi (99.6%), with only 0.4% being non-Bangladeshi. Overall, the prevalence of Premenstrual Tension Syndrome (PMTS) was 65.2% and 8.1% of participants met the criteria for PMDD (Table 1).

**Table 1:** Characteristics of the study participants (*n* = 273)

Variables	Frequency (*n*)	Percentages (%)
Age in years		
17–20	7	2.6
21–22	87	31.9
23–24	125	45.8
≥25	54	19.7
Mean ± SD	23.23 ± 1.76	
Age of menarche in years		
<12	33	12.1
≥12	240	87.9
Marital Status		
Single	239	87.5
Married	34	12.5
Nationality		
Local	0	
Bangladeshi	272	99.6
Non-Bangladeshi	1	0.4
PMT symptoms		
No	95	34.8
Mild	64	23.4
Moderate	92	33.7
Severe	22	8.1

Abbreviations: *PMT* Premenstrual tension, *SD* standard deviation

### Changes in psychological, behavioral and physical symptoms

**Fig. 1:**
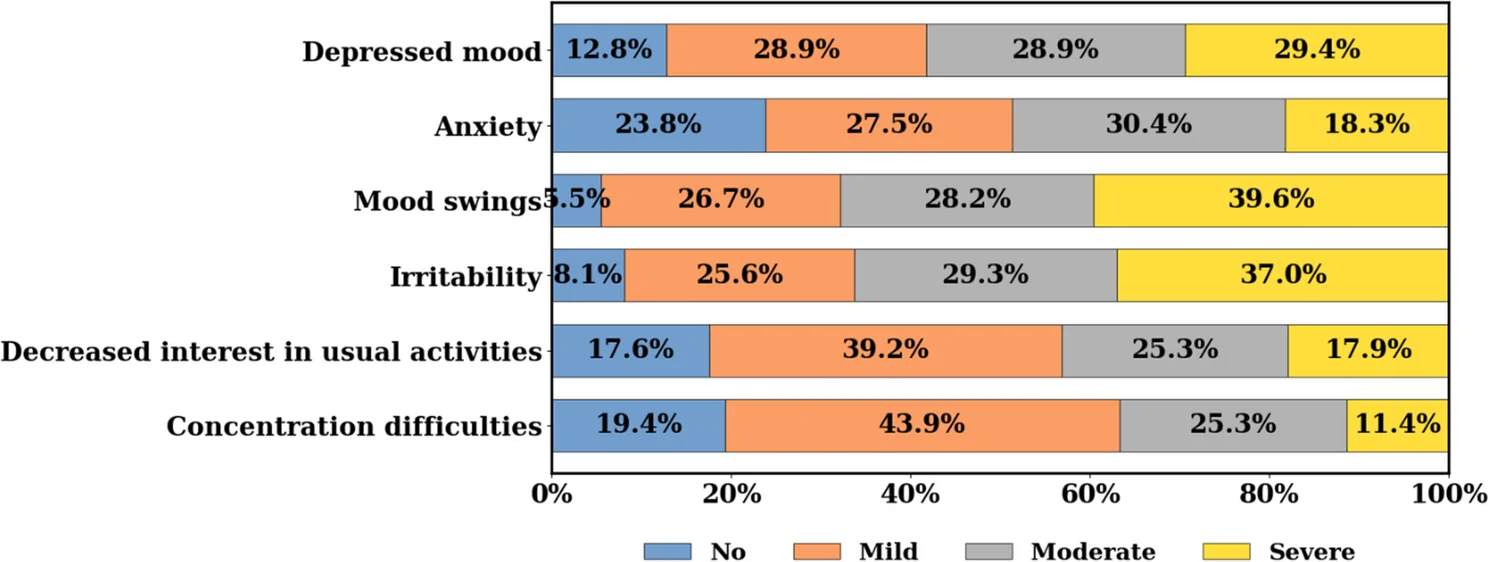
Variations in psychological symptoms associated with PMT syndrome among female participants

The distribution and intensity of six core psychological PMTS symptoms with respect to the four distinct severity patterns among female students (N = 273) during PMT syndrome are illustrated in (Fig. 1). Among all severity categories, mild symptoms are the most prevalent for both concentration difficulties and activities with 43.9% and 39.2% of participants, respectively. On the other hand, mood swings and irritability are the most severe symptoms with 39.6% and 37.0% of participants reporting ‘Severe’ intensity, respectively. Very few students reported having no symptoms of these (5.5% and 8.1%). Compared to other symptoms, anxiety showed the highest number of students with no symptoms (23.8%). Among those who experienced anxiety, most reported moderate symptoms (30.4%). Finally, depressed mood showed an unusual pattern, it was almost equally distributed across mild, moderate and severe categories (approximately 29% each). This equal split is different from all other symptoms.

**Fig. 2:**
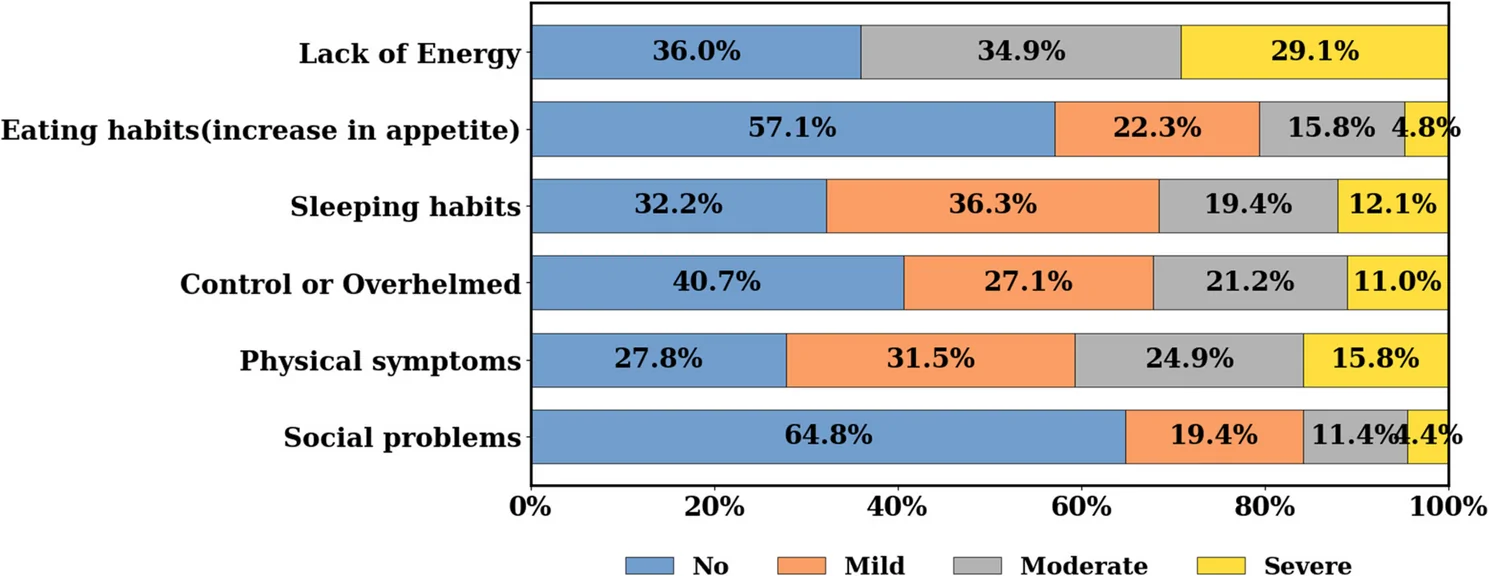
Variations in behavioral and physical symptoms associated with PMT syndrome among female participants

Considering behavioral and physical symptoms, (Fig. 2) shows that most students reported no symptoms for both social problems and increased appetite, with 64.8% and 57.1% of respondents, respectively. Only 4.4% of respondents reported severe social problems and 4.8% reported severe appetite changes. Psychical symptoms were experienced by 31.5% of respondents with mild symptoms and 24.9% with moderate symptoms, while sleep problems were mostly mild, affecting 36.3% of participants. Feeling overwhelmed was not reported by 40.7% of students, while the remaining students experienced mild, moderate, or severe symptoms. Lack of energy most frequently reported as severe (29.1%), while no students reported mild symptoms.

### Impact on academic performance

**Fig. 3:**
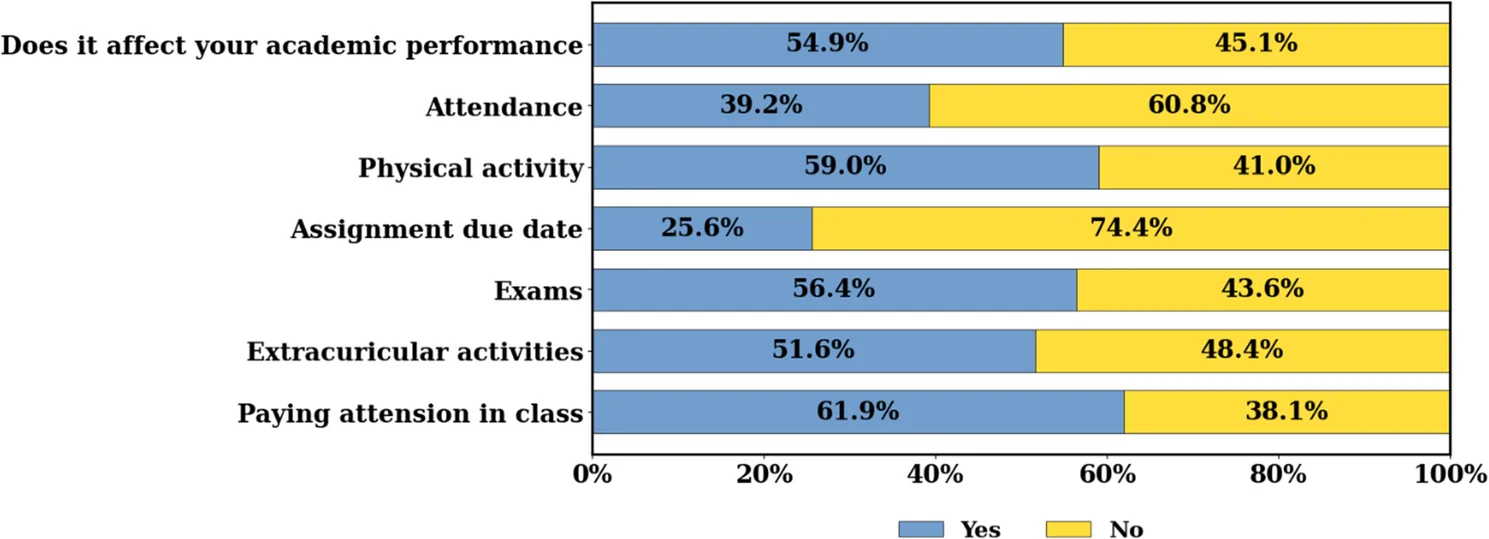
The impact of PMT syndrome on various aspects of the participants’ academic performance

Based on (Fig. 3), more than half of the students (54.9%) reported that PMT affected their overall academic performance. The most affected areas were concentration difficulties in class (61.9%), limitations in physical activities (59.0%), exam performance (56.4%), participation in extracurricular activities (51.6%), and difficulties in meeting assignment deadlines (25.6%).

Table 2 summarizes the association between premenstrual tension syndrome (PMT) and various aspects of academic performance, which was assessed using Chi-square tests. The analysis revealed that the most significant association exists between PMT and participation in extracurricular activities (p = 0.009). Specifically, 60.0% of students with PMT reported that they faced problems in extracurricular activities, which is much higher than that of students without PMT (43.5%). Secondly, a notable association was observed between PMT and its effect on examination performance (p = 0.012). Among students with PMT, 64.4% reported experiencing poorer exam performance, whereas 48.6% of students without PMT also reported similar problems. Thirdly, a strong association was found between PMT and attention in class (p = 0.013). Among students with PMT, 69.6% reported difficulty concentrating in class, which was comparatively higher than among students without PMT (54.3%). Furthermore, regarding physical activities, 65.2% of students with PMT reported difficulties, which is higher than those without PMT. This association approached statistical significance (p = 0.052). Finally, other factors such as class attendance (p = 0.102) and assignment deadlines (p = 0.176) did not show any statistically significant association with PMT.

**Table 2:** Association between the influence of PMT syndrome and academic performance among participants

Variables	PMT, *n* (%)		
	No (*n* = 138)	Yes (*n* = 135)	*p*-Value^a^
Effect on attendance			0.102
No	91 (65.9%)	75 (55.6%)	
Yes	47 (34.1%)	60 (44.4%)	
Effect on physical activities			0.052
No	65 (47.1%)	47 (34.8%)	
Yes	73 (52.9%)	88 (65.2%)	
Effect on assignments due dates			0.176
No	108 (78.3%)	95 (70.4%)	
Yes	30 (21.7%)	40 (29.6%)	
Effect on exams			0.012
No	71 (51.4%)	48 (35.6%)	
Yes	67 (48.6%)	87 (64.4%)	
Effect on extracurricular activities			0.009
No	78 (56.5%)	54 (40.0%)	
Yes	60 (43.5%)	81 (60.0%)	
Effect on paying attention in class			0.013
No	63 (45.7%)	41 (30.4%)	
Yes	75 (54.3%)	94 (69.6%)	

Abbreviation: *PMT* premenstrual tension

*p* value < 0.05 is significant

^a^Chi-square test

## Discussions

The present study aimed to determine the prevalence and severity of premenstrual tension (PMT) syndrome and its association with academic performance among female university students in Bangladesh. The findings revealed that 65.2% of participants experienced PMT symptoms ranging from mild to severe, while 8.1% met the criteria for Premenstrual Dysphoric Disorder (PMDD). In addition, PMT was significantly associated with academic impairment, particularly difficulty concentrating in class (69.6%) and reduced examination performance (64.4%).

The observed prevalence of PMT syndrome in this study (65.2%) is consistent with previously reported estimates among university students globally, which range between 20% and 90% [21–24]. A meta-analysis from China reported a pooled prevalence of 48% [25], while studies among female university and college students in India and Iran reported 51.4% and 68.9%, respectively [26, 27]. In the South Asian context, a study from Nepal reported a prevalence of 62.5% [28], which closely aligns with the findings of the present study. Variability in prevalence across studies may be attributed to differences in diagnostic criteria, assessment tools, cultural context, psychological stress levels, lifestyle factors, menstrual awareness, and reproductive characteristics [29–32].

The prevalence of PMDD (8.1%) in the present study falls within the global range of 3% to 8% [5], and is comparable to findings among female university students in Jordan (7.7%) [33], as well as in other comparable populations, including university-level students (8.67%) [34] and young women in Turkey (10.0%) [35]. Although methodological and population differences exist across studies, these findings indicate that PMDD is a consistent clinical concern among university students and similar populations. Given its position as the severe end of the premenstrual disorder spectrum, its presence among a notable proportion of students highlights the need for early identification and appropriate management strategies.

Notably, psychological symptoms were the most prominent in the present study. Mood swings (39.6% severe) and irritability (37.0%) were the leading severe manifestations. In contrast, physical symptoms were comparatively less severe (15.8%), although they still affected a considerable proportion of students. Among behavioral symptoms, lack of energy (29.1%) was also a notable concern.

Similarly, studies among college students in Bhavnagar, Gujarat, India [23] identified mood swings and irritability as predominant symptoms, which is consistent with the present findings. In addition, a study among university students in Monastir, Tunisia reported that psychological symptoms, including mood swings (70.7%) and irritability (71.3%), were among the most frequently observed premenstrual manifestations [36]. Moreover, irritability has been reported as one of the most common premenstrual symptoms in several developing-country settings, including Turkey, Lebanon, and Nigeria [37, 38].

Lack of energy was reported at moderate to severe levels by approximately 64% of participants. This finding is consistent with research from Gujarat, India, where fatigue/lack of energy was identified as one of the most commonly reported premenstrual symptoms (68.3%) and was associated with significantly impaired school/work efficiency and productivity (88.9%) [23]. Similarly, a study conducted in Casablanca, Morocco reported lethargy and excessive fatigability (lack of energy) as the most frequent premenstrual symptoms (91.6%) [39]. Comparable patterns have also been observed in developed countries, including Japan, Spain, and France, where lack of energy has consistently been identified among the most commonly reported premenstrual symptoms [37, 40].

Taken together, these findings indicate that psychological symptoms represent a consistent and prominent component of the premenstrual experience across diverse cultural and educational contexts. Although physical symptoms (15.8%) and social impairment (4.4%) were comparatively lower, affected students may maintain outward functional activity while experiencing substantial internal psychological distress.

Moreover, PMT demonstrated a significant negative impact on academic functioning. Specifically, 69.6% of students reported difficulty concentrating in class, 64.4% indicated adverse effects on examination performance, and 60.0% experienced difficulty participating in extracurricular activities.

These findings are consistent with previous evidence demonstrating the negative impact of premenstrual conditions on academic functioning among female university students. For instance, a meta-analysis among Ethiopian female students reported significant functional and academic impairment associated with premenstrual disorders [41]. Similar associations have been documented in studies from the United Arab Emirates (UAE) [1], Afghanistan [42], Jordan [43], and Brazil [44].

Likewise, previous research reported that although only a small proportion of students were absent from class (12%), a considerably higher proportion (32%) avoided social activities during the premenstrual period [24].

Overall, 54.9% of participants in the present study reported that their academic performance was adversely affected by premenstrual symptoms, indicating a substantial functional burden of PMT among female university students.

Regarding reproductive factors, most participants (87.9%) reported menarche at ≥ 12 years. A study among Chinese university students in Hong Kong reported that older age at menarche was significantly associated with a lower risk of PMDD [45]. However, the occurrence of PMDD in 8.1% of participants in the present study suggests that menstrual timing alone may not fully explain PMDD risk. Other factors, including lifestyle, psychological stress, and physical activity, may also contribute [46, 47].

Overall, these findings indicate that premenstrual tension syndrome among female university students is not merely a menstrual cycle-related phenomenon but a significant public health concern affecting academic performance, mental well-being, and social engagement.

Globally, evidence indicates a high prevalence of premenstrual symptoms. For instance, a systematic review and meta-analysis reported a pooled prevalence of 47.8% among women of reproductive age [48]. Similarly, a large meta-analysis conducted in China involving 108,178 participants reported a pooled prevalence of 48% [25].

In low- and middle-income countries such as Bangladesh, limited menstrual health awareness, social stigma, and restricted access to healthcare services may further exacerbate the burden of PMT among female university students [49, 50]. Therefore, the implementation of university-based awareness programs, mental health support services, and evidence-based policy interventions is both timely and essential.

### Strengths and limitations 

This study is the first systematic investigation of premenstrual tension (PMT) and its academic impact among female students at a leading university in southwestern Bangladesh, using a reliable DSM-5–based bilingual questionnaire (α = 0.878) to assess psychological, behavioral, and physical symptoms and their effects on academic outcomes, including examination performance, classroom attention, and co-curricular activities. This provides an important baseline for future research in Bangladesh and similar South Asian contexts.

The study applied strict inclusion and exclusion criteria to ensure that participants represented PMT-affected individuals only, and a sample size of 273 ensured adequate statistical reliability and representativeness within the study setting.

However, several limitations should be acknowledged. First, the cross-sectional design limits the ability to establish causal relationships between PMT and academic performance; therefore, only associations can be inferred. Second, the study relied on self-reported data, which may introduce recall bias and social desirability bias, particularly due to the sensitive nature of menstrual health reporting. Third, generalizability is limited as the study included only residential female students from a single university. Additionally, potential confounding factors such as diet, physical activity, and social support were not assessed, which may influence PMT severity and coping behavior.

## Conclusions 

This study shows that premenstrual tension (PMT) is very common among female university students in Bangladesh and strongly affects their academic life. Mood swings and irritability were the most severe symptoms, linked to poor attention in class, lower exam results, and reduced participation in extracurricular activities. PMT is more than a menstrual issue; it impacts mental health, academics, and social life. Universities should raise awareness, provide mental health support, and adopt evidence-based policies. This study provides a foundation for future research, where larger studies with more data and longer follow-up can better clarify PMT’s impact on academic performance.

## Supplementary information

Supplementary Material 1.

## Data Availability

Data are available from the corresponding author upon reasonable request.

## Abbreviations

PMT: Premenstrual Tension
DSM-5: Diagnostic and Statistical Manual of Mental Disorder, 5th Edition
SPSS: Statistical Package for the Social Sciences
PMDD: Premenstrual Dysphoric Disorder
SSRIs: Selective Serotonin Reuptake Inhibitors
PMS: Premenstrual Syndrome
α: Cronbach’s Alpha
PMTS: Premenstrual Tension Syndrome
SD: Standard Deviation
n: Frequency
IU: Islamic University
