# Supplementary material for: Prevalence and severity of premenstrual tension syndrome and its impact on academic performance among female university students in Bangladesh: a cross-sectional study
Source: BMC Public Health. 2026 Jul 2;26:2833. doi: 10.1186/s12889-026-28124-3 (PMC13644285; doi:10.1186/s12889-026-28124-3)
Supplement: Supplementary file 1 [file 12889_2026_28124_MOESM1_ESM.pdf]

# Research Questionnaire

## Impact of Premenstrual Tension Syndrome on Academic Performance

This questionnaire has been designed to assess the Prevalence and severity of premenstrual tension (PMT) symptoms and its impact on academic performance among female university students in Bangladesh. Your honest responses will help us better understand this relationship. Please note that all responses will be kept strictly confidential.

## Part I: Demographic Information

**1. What is your age?**

□ 17-20 years □ 21-22 years □ 23-24 years □ 25 years or older

**2. What is your marital status?**

□ Single □ Married

**3. What is your nationality?**

□ Local □ Bangladeshi □ Non-Bangladeshi

**4. Year of Study:**

□ First Year □ Second Year □ Third Year □ Fourth Year □ Fifth Year

## Part II: Menstrual History

**5. At what age did you experience your first period (menarche)?**

□ Before 12 years □ 12 years or older

**6. Do you experience premenstrual tension (PMT) syndrome?**

□ Yes □ No

**7. How severe are your PMT symptoms?**

□ No □ Mild □ Moderate □ Severe

## Part III: Premenstrual syndrome (PMS) Symptoms and Severity

*During the week before your menstrual period, how would you rate the following symptoms?*

*Hints: No (0), Mild (1), Moderate (2), Severe (3)*

| Symptom | No (0) | Mild (1) | Moderate (2) | Severe (3) |
|---|---|---|---|---|
| **Psychological Symptoms** | | | | |
| 8. Concentration difficulties | ○ | ○ | ○ | ○ |
| 9. Decreased interest in usual activities | ○ | ○ | ○ | ○ |
| 10. Irritability | ○ | ○ | ○ | ○ |
| 11. Mood swings | ○ | ○ | ○ | ○ |
| 12. Anxiety | ○ | ○ | ○ | ○ |
| 13. Depressed mood | ○ | ○ | ○ | ○ |
| **Physical & Behavioral Symptoms** | | | | |
| 14. Physical symptoms (breast tenderness, bloating, etc.) | ○ | ○ | ○ | ○ |
| 15. Excessive appetite | ○ | ○ | ○ | ○ |
| 16. Changes in sleep patterns | ○ | ○ | ○ | ○ |
| 17. Lack of energy or motivation | ○ | ○ | ○ | ○ |
| 18. Feeling overwhelmed or out of control | ○ | ○ | ○ | ○ |
| 19. Social problems | ○ | ○ | ○ | ○ |

## Part IV: Impact on Academic Performance

*During the week before your menstrual period, how do PMS symptoms affect the following academic activities? (Please answer Yes or No for each question)*

**20. Does it negatively affect your class attendance?**

□ Yes □ No

**21. Does it cause any limitation in participating in physical activities?**

□ Yes □ No

**22. Do you have difficulty submitting assignments on time?**

□ Yes □ No

**23. Does it affect your exam performance?**

□ Yes □ No

**24. Does it hinder your participation in extracurricular activities?**

□ Yes □ No

**25. Do you have difficulty concentrating in class?**

□ Yes □ No

**26. Does it affect your academic performance?**

□ Yes □ No

## Part V: Statistical Analysis Information

**27. Would you like to participate in a follow-up study?**

□ Yes □ No

**28. Do you give consent for your responses to be used anonymously for research purposes?**

□ Yes □ No

---

**Thank you for taking the time to complete this questionnaire!**

Your participation is valuable for understanding the prevalence and severity of premenstrual tension (PMT) symptoms and their impact on academic performance.

**Confidentiality Assurance:** All responses will remain strictly confidential and will be used for research purposes only. No individual responses will be identified or disclosed.
